# Use of Rescue Flaps in the Reconstruction of Anterior Skull Base Defects

**DOI:** 10.7759/cureus.46896

**Published:** 2023-10-12

**Authors:** Drishti Singh, Gajanan Pisulkar

**Affiliations:** 1 Surgery, Jawaharlal Nehru Medical College , Datta Meghe Institute of Higher Education and Research, Wardha, IND; 2 Orthopaedic Surgery, Jawaharlal Nehru Medical College , Datta Meghe Institute of Higher Education and Research, Wardha, IND

**Keywords:** reconstruction of skull base, endoscopic endonasal skull base surgery, transsphenoidal approach, rescue flap, anterior and lateral skull base surgery

## Abstract

Several traumatic and non-traumatic defects in the anterior base of the skull require incessant reconstruction to stop the leakage of cerebrospinal fluid (CSF). Reconstruction of these defects at the earliest is essential to achieve an uncomplicated recovery. Various innovations in surgical procedures are seen contemporarily in reconstructing the weaknesses in the anterior part of the skull base. Multilayer grafting techniques successfully repair minor dural defects, while significant dural defects require pedicled vascularized grafts for reconstruction. Using nasoseptal flaps (NSFs) has drastically lowered the instances of CSF leaks in significant dural defects. The rescue flap is an advancement in the approach of the NSF, which was discovered in 2011. This flap is made in a downward direction with the formation of a posterior superior incision so that it does not interfere with the mucosal flap. A small incision is made at the ostium of the sphenoid bone, which is brought into the anterior aspect of the superior nasal septum. The mucosa is elevated inferiorly through the ostium of the sphenoid bone, so some septal branch of the sphenopalatine artery is preserved. In this way, the vascular supply is protected. However, in cases of CSF leak during operations, this rescue flap is reverted into an atypical and standard NSF for reconstructing the base of the skull. This rescue flap technique gives a binaural approach to sella in a way that does not compromise the pedicle during tumor removal. This rescue flap significantly decreases the duration of care in the post-operative phase and improves the cost efficiency of the surgery by avoiding donor site morbidity.

## Introduction and background

The skull base is a three-dimensional structure situated in an exigent location in the human body and requires an incredible understanding of human anatomy in its reconstruction approach. Numerous traumatic and non-traumatic factors can cause defects in the skull base. Among the traumatic causes of skull base defects, the most common is surgical trauma, followed by damage caused by craniofacial traumas. High intracranial pressure and malignant and some benign neoplasms are the non-traumatic causes of skull base defects. Rarely, infections and radiotherapy can also cause skull base defects. These pathologies can cause spontaneous leakage of cerebrospinal fluid (CSF) [1]. It is crucial to reconstruct the skull base to achieve an uneventful post-operative course. In the last decade, the contemporary world has seen immense surgical evolution in anterior skull base reconstruction. The emergence of endoscopic endonasal approaches led to advanced and skilled instrumentation in the excision of intramural and extramural lesions of the skull base, and reconstruction was accomplished. Multilayer grafting techniques have shown a sky-high success rate in the repair of minor dural defects. On the other hand, significant dural defects with a significant leak of CSF require pedicled vascularized grafts [2]. Skull base reconstruction provides a tight demarcation between the intracranial and extracranial contents to return its form and function.

The use of vascularized tissues in the reconstruction of significant defects in the base of the skull has proved to be immensely useful for separating paranasal sinuses from the contents of the skull following endoscopic endonasal approaches. One technique that has revolutionized anterior skull base surgeries is the nasoseptal flap (NSF), a local pedicle-based flap based on the nasoseptal artery [3]. The rescue flap works by harvesting the superior and posterior parts of the flap so that its pedicle is protected and the face of the sphenoid is exposed during the approach world has, however, broadened the use of these flaps. Several variants of NSFs have come into the picture. These include NSF rescue flaps, posterior NSFs, Janus flaps, posterior pedicled inferior turbinate flaps, middle turbinate flaps, etc. However, a few complications have been identified while using the flap, which include epistaxis, asymptomatic synechiae, adverse effects on craniofacial development in children, otalgia, and hyposmia [4].

## Review

Methodology

**Figure 1:**
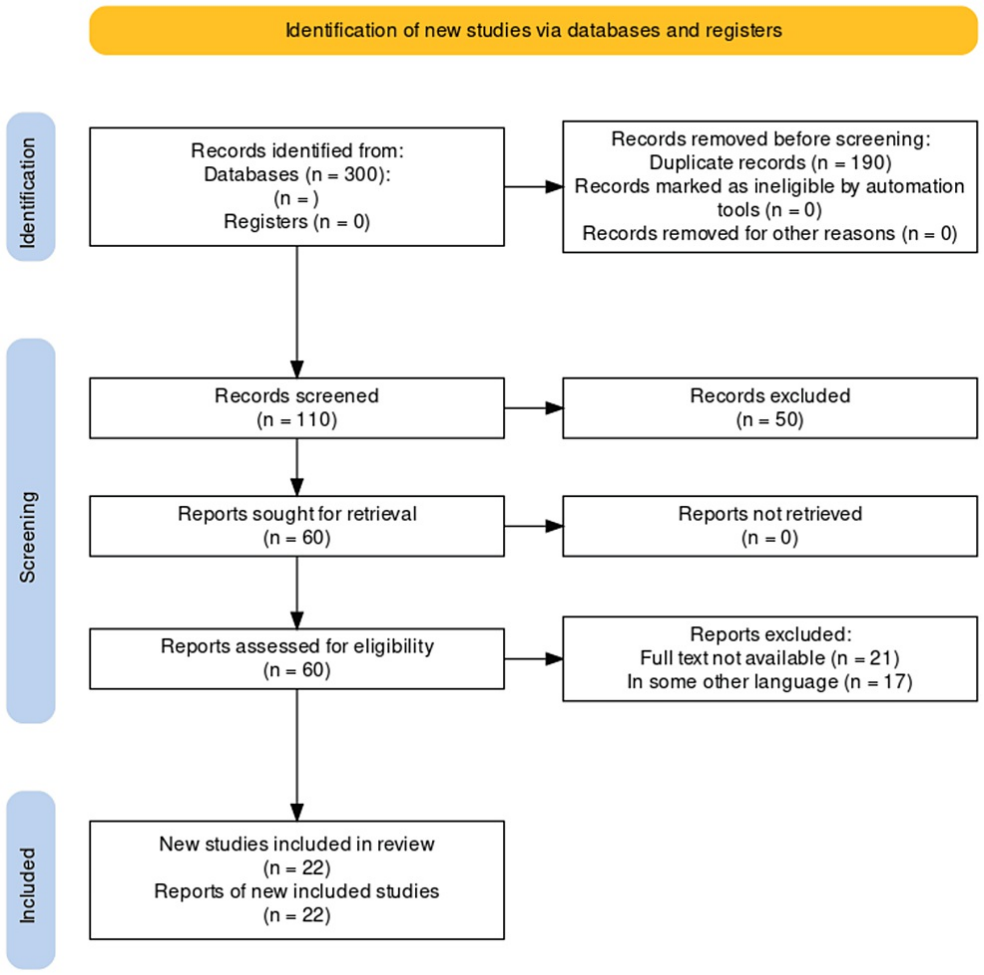
PRISMA diagram for reconstruction of the anterior skull base using rescue flaps Credit: Author PRISMA: Preferred Reporting Items for Systematic Reviews and Meta-Analyses

A literature search was conducted to comprehensively review the use of the rescue flap technique for reconstruction of anterior skull base defects. Multiple electronic databases including PubMed, MEDLINE, Embase, and Google Scholar were searched using the following keywords and combinations anterior skull base surgery, rescue flaps, nasoseptal flaps, and endoscopic endonasal approach. The search encompassed articles published from 2002 to 2022. In addition to electronic database searches, reference lists of relevant articles and review papers were manually screened to identify additional studies. The inclusion criteria involved selecting observational studies, experimental studies, systematic reviews, and meta-analyses that enlisted various outcomes of the surgeries performed using rescue flaps. Studies with human participants were included while focusing solely on reconstruction using rescue flaps. Only peer-reviewed, published articles were considered for inclusion. Two independent reviewers assessed the titles, abstracts, and full-text articles for eligibility, with any discrepancies resolved through discussion and consensus. The comprehensive literature search aimed to ensure the inclusion of relevant studies and provide a thorough analysis of the use of rescue flaps in the reconstruction of anterior skull base defects. Figure 1 describes the selection process of articles used in our study. 

Anterior skull base

The base of the skull comprises the middle, anterior, and posterior fossa. The boundary of the anterior skull base constitutes crista galli and cribriform plate medially, frontal bone laterally, and lesser wings of sphenoid bone posteriorly. Pathological lesions extending from the nose and paranasal sinuses are the most common cause of defects in the anterior part of the base of the skull. Computed tomography (CT)scans, magnetic resonance imaging (MRI), and post-intravenous gadolinium MRI scans are performed to note the provisional lesions. Other tools for anterior skull base imaging include post-contrast T1W and T-2 weighted sequences [5,6]. Lesions affecting the anterior skull base from below are benign tumors like secondary mucocele, osteomalacia, fibrous dysplasia, angiofibroma, and inverted papilloma [7]. Malignant neoplasms affecting the skull base include primary sinonasal cancers, lymphoma, rhabdomyosarcoma, and salivary gland tumors. Congenital conditions can also cause anterior skull base defects [8]. Among the acquired causes are traumatic or iatrogenic injuries, olfactory groove meningioma, and encephalomalacia of the structures in the inferior aspect of the frontal lobe, which also result in behavioral personality changes. Reconstruction of the base of anterior skull base defects is based on their location and size. Pedicled NSFs are the most efficient in correcting significant defects of the skull base [9]. Rescue flaps, or the nasoseptal rescue flap, were introduced by modifying the NSF in 2011. The NSF rescue flap is a modified variant of the prototypical NSF. In rescue flaps, only a posterior superior incision is made in the initial stages of the operation [10]. The flap is made inferiorly downward from the posterosuperior septum and sphenoid face, reaching the level of sphenoid sinus floor. In this, a small incision is made at the ostium of the sphenoid bone and brought anteriorly to the superior nasal septum. Through this ostium, the mucosa is pushed upward inferiorly, preserving the posterior septal branch of the sphenopalatine artery. In this way, the vascular supply is protected. This artery is responsible for supplying blood to the NSF. As the sphenoid is opened to reach the sella, NSFs from both sides can be harvested [11]. This technique allows the bimanual and binaural approach to access the sella in a way that does not compromise the pedicle during tumor removal. The rescue flap is made before tumor removal to reduce pedicle flap damage and estimate the size of the pedicle flap required post-tumor removal [12]. However, in cases of CSF leak during operations, this rescue flap is reverted to a regular NSF for reconstructing the base of the skull.

*Surgical Technique* 

**Figure 2:**
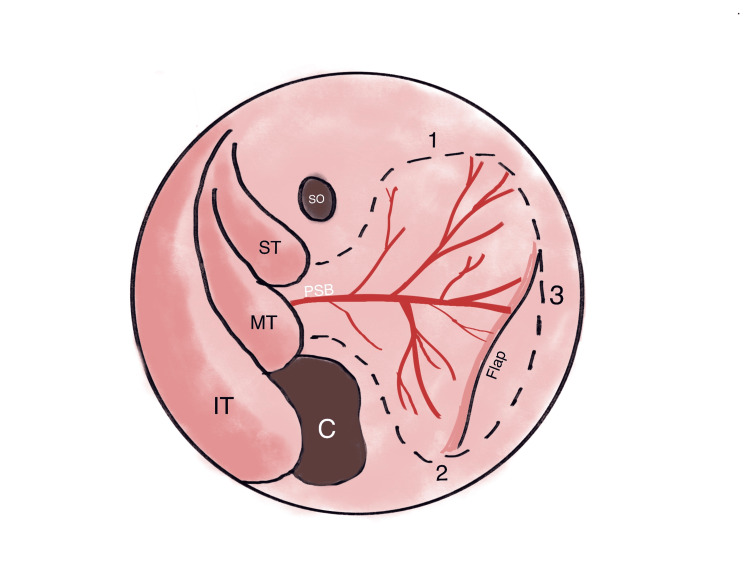
The sketch depicts the outline of a nasoseptal flap in the nasal cavity when viewed under endoscopy. The superior (1), inferior (2), and anterior (3) incisions of the nasoseptal flaps. Laterally extended inferior incision can be used to maximize the area of the graft. The image is created by Drishti Singh ST: Superior turbinate, MT: Middle turbinate, IT: Inferior turbinate, C: Choana, SO: Sphenoid ostium [3]

In cases with no suspected CSF leak, rescue flaps are made use of. The patient is made to lie supine with an elevated trunk, and the patient’s head is turned in the surgeon’s direction. Decongestion of the nasal pathway is done, followed by fracturing the inferior turbinate along with one of the middle turbinates to visualize the position of the transsphenoidal opening. This is done to improve bimanual technique and visualization during the pituitary approach. A transverse incision is done with a monopolar tip of the needle on the side of the middle turbinate at the ostium of the sphenoid bone. This incision is anteriorly continued into the nasal septum and medially to the sphenoid rostrum through the sagittal plane. A mucosal flap is structured using an elevator by raising the mucosa below the incision in a submucoperichondrial/subperiosteal fashion. A wide sphenoidotomy is performed, followed by posterosuperior septectomy of the nose. Preservation of the septum and sphenoid facial mucosa is done. Protection of the rescue flap, pedicle, and proximal part of the NSF is brought about by raising the flap. The previously made rescue flap is preserved, and ipsilateral sphenoidotomy is performed anterior to the pedicle of the rescue flap. After completing bilateral sphenoidotomies, downward displacement of the flap is done. In a rescue flap, the sphenoid ostium is the site of one horizontal incision, so the posterior nasal septal artery is spared. Septal perforation in the rear part is created by mucosal removal. The mucosa is preserved, but it is not primarily closed or reconstructed. In cases where CSF leaks occur, repositioning the posterior part of the mucosal flap is performed [13-16]. Figure 2 depicts the outline of an NSF when viewed under endoscopy.

Few studies are being performed on the rescue flap approach in the reconstruction of anterior skull base defects that are said to have eliminated donor site morbidity in patients who do not encounter a leak in CSF during the surgical procedure. A study was conducted on five preserved and three fresh human specimens, injected with red and blue silicone into the internal carotid artery and jugular vein. The rescue flap surgery was performed in cases where a CSF leak was unexpected. Photographs were clicked with 0-degree and 30-degree endoscopes comprising rod lenses connected to a monitor and camera. However, if significant defects were encountered or a leak in CSF occurred, the rescue flap was entirely harvested. The rescue flap gave biannual and binaural access to the sella without compromising the pedicels. The injected cadavers harvested the rescue flap without interfering with or compromising biannual stellar exposure. A full flap was raised in all the specimens without disrupting the sphenopalatine artery. This technique decreased donor site morbidity and time spent in postoperative care and improved cost efficiency [16]. On performing a retrospective study, which included 125 trans-sphenoidal approached cases in which the nasoseptal rescue flap (NSRF) technique was implicated for surgery at the Cleveland Clinic. It was observed that only 16 per cent of patients had to undergo a conversion of NSRF to a full nasoseptal flap. The reasons attributed to this were an unexpectedly high or low flow cerebrospinal fluid leak and an exposed internal carotid artery. No CSF leak or ischemia of the flap was seen in the patients who underwent full NSF-raised surgery. The study concluded by asserting that NSRF provides the flexibility and reliability of vascularized reconstruction [11]. In a cross-sectional study performed in the Otorhinolaryngology department of a tertiary care hospital in central India on 20 patients, the outcome of rescue flaps, CSF leak during surgery, and donor site morbidity were evaluated. The patients underwent a thorough nasopharyngeal examination, anterior rhinoscopy, posterior rhinoscopy, and diagnostic nasal endoscopy before surgery. These patients were then operated on by using endonasal endoscopic surgery for the correction of skull base lesions through the creation of a bilateral rescue flap before the excision of the tumor. The rescue flap was converted into NSFs in cases of significant defects or CSF leaks. Pack removal was done after three to five days, and follow-up was taken on the fifth day, one month, and three months after surgery. This study was done with the hope that it might bring precision to surgical interventions. The flap was elevated from the mucoperiosteum, which lined the posterior part of the nasal septum. Thus, the anterior septal cartilage, which lay anteriorly, was not denuded. Raising such flaps avoids postoperative morbidity and decreases operative time and wound complications [16].

The neurosurgery department of the Shandong University of Qilu Hospital used the rescue flap for a transsphenoidal approach in resectioning adenoma on 113 patients, which gave satisfying results. Twenty-seven cases of CSF leakage during the operation were documented. Among these, 24 cases were of low-flow CSF leaks, and three were of high-flow CSF leaks. The nasoseptal “rescue” flap was reverted to the nasal septum flap in 23 patients due to the formation of a CSF fistula. The olfactory decline was seen in 17 cases. One case presented with epistaxis, and one with cerebrospinal rhinorrhea after surgery. The nasoseptal approach of the “rescue” flap technique decreases the chances of fatal complications. It was found that the rescue flap provided a steady and efficient way to invade the sphenoid sinus surgically and paved the way for constructing a nasal septal pedicled mucosal flap for any future occurrence of cerebrospinal fluid leakage. This case highlighted the fact that no fluke was encountered during the operation of pituitary adenoma under neuro endoscope; a rescue flap could be used to repair every single leakage. Reconstruction of the floor of the sella, if a CSF fistula is encountered while operating, could be successfully done by these rescue flaps without prolonging the time taken for the surgery [17]. Another experiment was conducted by Kim et al. to evaluate the efficiency and usefulness of rescue flaps elevation in an endoscopic endonasal transsphenoidal approach (EETSA) [18]. The study was conducted between February 2009 and June 2012 on 92 patients who underwent EETSA with bilateral modified nasoseptal rescue flaps elevation. Specific preoperative examinations like nasal obstruction symptom elevation (NOSE), sinonasal outcome test (SNOT), etc. were performed on the patients. After the surgery, a CSF leak was reported in 17 of these patients, three out of whom underwent conversion of the rescue flap into a conventional NSF. Re operation due to CSF leak was not performed in any patient. The NOSE and SNOT scores appeared the same statistically even after the surgical procedure. Thus, it was concluded that bilateral modified nasoseptal rescue flaps elevation exposed the stellar floor correctly, thereby also preserving the sphenopalatine artery. It was also instrumental in minimizing post-operative complications [18]. In a study conducted at the University of North Carolina-Chapel Hill, it was observed that the nasoseptal rescue flap provided complete reconstruction in dural anterior skull base defects in cases where CSF leak occurs during surgery and reduction in morbidity of donor site in those cases where the leak is not encountered. The study was performed on 26 patients by harvesting nasoseptal rescue flaps, out of whom only seven percent were in actual need of a rescue flap for skull base reconstruction due to intra-operative CSF leak. One patient presented with a high CSF leak, and six presented with low CSF leaks. After a mean follow-up time of six months, it was observed that none of the patients presented with septal perforation or CSF leak. A 100% success rate was seen in those seven patients with rescue flap utilization [19].

The endoscopic endonasal skull base surgery (EESBS) was performed using the sigmoid incision rescue flap, reducing the invasiveness and providing an efficient surgical corridor for EESBS. A nasoseptal flap is used to manage postoperative CSF leaks after removing the tumor. To preserve the pedicle, the nasoseptal is to be elevated before adenoidectomy and septectomy. In cases where a CSF leak is absent, a rescue flap technique is used. A new sigmoid incision (S-I) rescue flap method is devised, giving a broader view of the sphenoid rostrum. Only two complications were documented in a study conducted by Ozawa et al. on 19 patients who underwent EESBS with an S-I rescue flap to remove the tumor. These included CSF leak and epistaxis, which were incessantly corrected. The S-I rescue flap was observed to be instrumental in reducing invasiveness and paving the way for performing EESBS [20]. The pericranial rescue flap covers both anterior skull base defects and seller defects through the endonasal approach. Dissections were performed on 12 cadavers to justify the efficiency of the lateral pericranial rescue flap. An incision is made on the eyebrow from the middle to the lateral side. The galea layer was separated from the pericranium, and using an endoscope, the periosteal flap was raised. The pericranial flap was inserted through the extradural or intradural route, a burr-hole is created in the supraorbital bone. It was observed that the average dimensions of this flap were 11.5 cm to 3.2 cm, which was large enough to reconstruct and repair the defects of the anterior skull base. These minimally invasive pericranial flaps also resolved the issue of CSF leakage [21]. Table 1 depicts the outcome of various surgeries performed using the rescue flap techniques.

**Table 1:** Outcomes of various surgeries performed by using rescue flaps in reconstruction of anterior skull base defects The table is created by the author.

Various surgeries performed using “Rescue flaps”	Outcomes of the surgery
Retrospective study at the Cleveland Clinic on 125 transsphenoidal cases [11]	Conversion of a nasoseptal rescue flap to a full nasoseptal flap in 16 percent of the patients
Cross sectional study in the Otorhinolaryngology department of a tertiary-care hospital in central India on 20 patients [16]	Rescue flaps were converted into nasoseptal flaps in cases of large defects or cerebrospinal fluid leaks along with pack removal after three to five days.
The department of Neurosurgery of the Shandong University of Qilu Hospital on 113 patients [17]	Twenty-seven cases of intra-operative cerebrospinal fluid leaks, 23 patients underwent conversion of Rescue flaps to nasal septum flaps due to formation of a fistula, olfactory decline was observed in 17 cases, one case of epistaxis and one case of cerebrospinal rhinorrhea after surgery.
Experiment conducted by Kim et al. which evaluated the efficiency of rescue flaps elevation in an endoscopic endonasal transsphenoidal approach on 92 patients [18]	Cerebrospinal fluid leak was reported in 17 of 92 patients, conversion of rescue flap to a conventional nasoseptal flap in three patients.
Study conducted at the University of North Carolina-Chapel Hill on 26 patients [19]	One patient presented with high cerebrospinal fluid leak, six patients presented with low-cerebrospinal fluid leaks.
Study conducted by Ozawa et al. using a sigmoid incision rescue flap on 19 patients [20]	Cerebrospinal fluid and epistaxis in two cases

Various other techniques have also been used to reconstruct the defects of the anterior part of the skull. These include the free grafts and pedicled flaps, pedicled extranasal flaps, palatal flaps, pericranial flaps, facial buccinator flaps, temporoparietal flaps, etc. Skull base surgeries have been revolutionized recently using the rescue flap approach. However, certain complications still arise due to the use of these flaps. These flaps can cause the displacement of the olfactory epithelium, sphenoid sinus obstruction, and nose crusting due to the pedicle's orientation. Other complications include epistaxis, CSF leak, and asymptomatic synechiae. The indications for this flap are increasing manifolds, and more surgeons around the globe are utilizing it [22].

## Conclusions

Skull-base defects are caused by numerous non-traumatic as well as traumatic causes. Non-surgical trauma is the most common traumatic defect. Non-traumatic causes include neoplasms of malignant origin, infections, and radiotherapy, which results in cerebrospinal leakage. Hence, it is imperative to reconstruct the skull base defects for an uncomplicated postoperative course. The use of nasoseptal flaps has circumstantially decreased CSF leak rates during endoscopic anterior skull base surgeries. In cases of pituitary tumors where a CSF leak is highly unlikely, we can make use of a “rescue flap” approach by harvesting the posterior and superior part of the flap to protect the pedicle and provide access to the sphenoid during the surgery. The flap gives bimanual and binaural reach to the sella without compromising the pedicle in removing the tumor and sphenoidotomies. This technique has been instrumental in minimizing morbidity at the donor sites in patients with no CSF leak during operations.

## References

[1] Reyes C, Mason E, Solares CA (2014). Panorama of reconstruction of skull base defects: from traditional open to endonasal endoscopic approaches, from free grafts to microvascular flaps. Int Arch Otorhinolaryngol.

[2] Hachem RA, Elkhatib A, Beer-Furlan A, Prevedello D, Carrau R (2016). Reconstructive techniques in skull base surgery after resection of malignant lesions: a wide array of choices. Curr Opin Otolaryngol Head Neck Surg.

[3] Rivera-Serrano CM, Snyderman CH, Gardner P (2011). Nasoseptal "rescue" flap: a novel modification of the nasoseptal flap technique for pituitary surgery. Laryngoscope.

[4] Moon JH, Kim EH, Kim SH (2019). Various modifications of a vascularized nasoseptal flap for repair of extensive skull base dural defects. J Neurosurg.

[5] Francies O, Makalanda L, Paraskevopolous D, Adams A (2018). Imaging review of the anterior skull base. Acta Radiol Open.

[6] Schmalfuss IM (2018). Imaging of endoscopic approaches to the anterior and central skull base. Clin Radiol.

[7] Snyderman CH, Lavigne P (2020). Benign tumors of the anterior cranial base. Adv Otorhinolaryngol.

[8] Hoffmann TK, Scheithauer MO, Sommer F, Lindemann J, Haberl EJ, Friebe-Hoffmann U, Theodoraki MN (2017). Surgery of anterior skull base lesions in children. Ann Otol Rhinol Laryngol.

[9] Bernal-Sprekelsen M, Rioja E, Enseñat J, Enriquez K, Viscovich L, Agredo-Lemos FE, Alobid I (2014). Management of anterior skull base defect depending on its size and location. Biomed Res Int.

[10] Gutierrez WR, Bennion DM, Walsh JE, Owen SR (2020). Vascular pedicled flaps for skull base defect reconstruction. Laryngoscope Investig Otolaryngol.

[11] Cappello ZJ, Tang DM, Roxbury CR (2020). Utility of the nasoseptal "rescue" flap approach: analysis of 125 consecutive patients and implications for routine transsphenoidal surgery. Am J Rhinol Allergy.

[12] Murakami D, Kuga D, Miyamoto Y (2021). A pedicled posterior septal-nasal floor flap and a novel rescue flap for skull base reconstruction. World Neurosurg.

[13] Benzer M, Biceroglu H, Ates MS (2019). Comparison between rescue flap and double flap technique. J Neurol Surg B Skull Base.

[14] Kassam AB, Thomas A, Carrau RL (2008). Endoscopic reconstruction of the cranial base using a pedicled nasoseptal flap. Neurosurgery.

[15] Hadad G, Bassagasteguy L, Carrau RL, Mataza JC, Kassam A, Snyderman CH, Mintz A (2006). A novel reconstructive technique after endoscopic expanded endonasal approaches: vascular pedicle nasoseptal flap. Laryngoscope.

[16] Saini A, Jain S, Deshkar P (2021). Utility of rescue flap in the reconstruction of skull base defects following transnasal endoscopic excision of sellar/supra-sellar lesions. J Pharm Res int.

[17] Xing M, Lv W, Wang J (2021). Sellar floor bone flap with a pedicled nasoseptal flap in endoscopic trans nasal pituitary adenoma surgery. J Craniofac Surg.

[18] Kim BY, Shin JH, Kang SG (2013). Bilateral modified nasoseptal "rescue" flaps in the endoscopic endonasal transsphenoidal approach. Laryngoscope.

[19] Rawal RB, Kimple AJ, Dugar DR, Zanation AM (2012). Minimizing morbidity in endoscopic pituitary surgery: outcomes of the novel nasoseptal rescue flap technique. Otolaryngol Head Neck Surg.

[20] Ozawa H, Tomita T, Watanabe Y (2016). Sigmoid incision rescue nasoseptal flap technique for endoscopic endonasal skull base surgery. Acta Otolaryngol.

[21] Jang CK, Park SJ, Kim EH (2022). Pedicled frontal periosteal rescue flap via eyebrow incision for skull base reconstruction (SevEN-002). BMC Surg.

[22] Tang IP, Carrau RL, Otto BA (2015). Technical nuances of commonly used vascularised flaps for skull base reconstruction. J Laryngol Otol.

